# A Dental Law in the District of Columbia

**Published:** 1892-07

**Authors:** 


### A Dental Law in the District of Columbia.

For two years or more an effort has been put forth for securing a law regulating the practice of dentistry in the District of Columbia. The effort has been mainly on the part of a few dentists in Washington City. It is true they have had some help from outside, but the burden of the work has been upon their shoulders. Congress was very deliberate in the consideration and enactment of this law, but has at last passed a very good act; it is briefer than many of our State laws, but it embraces all the leading points that are now regarded as important for such a law. [Ed.

An Act for the regulation of the practice of dentistry in the District of Columbia, and for the protection of the people from empiricism in relation thereto.

*Be it enacted by the Senate and House of Representatives of the United States of America in Congress assembled*, That it shall be unlawful for any person to practice dentistry in the District of Columbia unless such person shall register with the health officer in compliance with the requirements hereinafter provided.

Sec. 2. That a board to carry out the purposes of this act is hereby created, to be known as the board of dental examiners, to consist of five reputable dentists resident of and for three years last before appointment actively engaged in the practice of dentistry in the District of Columbia, to be appointed by the

Commissioners of said District for terms of five years and until their successors are appointed: *Provided*, That the first five appointments shall be made for terms of one, two, three, four, and five years, respectively. A majority of said board shall constitute a quorum. Vacancies occurring in said board shall be filled by appointment of eligible persons for unexpired terms.

SEC. 3. That it shall be the duty of the board of dental examiners, first, to organize by electing one of their number president and one secretary, to provide necessary books and blank forms, and publicly announce the requirements of this act and the time, place and means of complying with its provisions within thirty days from its passage; second, to promptly certify to the health officer for registration all who are engaged in the practice of dentistry in said district at the time of passage of this act who apply therefor; third, to test the fitness and pass upon the qualification of persons desiring to commence the practice of dentistry in said District after the passage of this act and certify to the health officer for registration such as prove, under examination in theory and practice of dentistry, qualified in the judgment of the board to practice dentistry in said District; fourth, to report immediately information of any violation of this act, and, annually, the transactions of the board to the Commissioners of the District of Columbia: *Provided*, That all graduates of dental colleges which require a three years' course of study shall be entitled to certificates upon payment of the certification fee and without examination as to their qualifications.

SEC. 4. That it shall be the duty of every person practicing dentistry in said District at the time of the passage of this act to make application to said board, in form prescribed by said board, for certification, and present the certificates thus obtained for registration to the health officer within sixty days from the passage of this act. Every such person so registering may continue to practice without incurring the penalties of this act.

SEC. 5. That persons desiring to commence the practice of dentistry in said District after the passage of this act shall first obtain a certificate of qualification from the board of dental examiners, granted under authority conferred upon said board by section three of this act, and present the same to the health officer for registration.

Sec. 6. That it shall be the duty of the health officer to register all persons presenting certificates from said board in a book kept for this purpose, and indorse upon each certificate the fact and date of such registration.

Sec. 7. That certificates issued and indorsed under the provisions of this act shall be evidence of the right of the person to whom granted to practice under this act.

Sec. 8. That anyone who shall practice or attempt to practice dentistry in the said District without having complied with the provisions of this act shall be deemed guilty of a misdemeanor, and, upon conviction thereof, shall be fined not less than fifty nor more than two hundred dollars, and in default of payment of such fine shall be imprisoned not less than thirty nor more than ninety days, said fines, when collected to be paid into the Treasury of the United States, to the credit of the District of Columbia: *Provided*, That nothing in this act shall be construed to interfere with physicians in the discharge of their professional duties, nor with students pursuing a regular uninterrupted dental college course or in bona fide pupilage with a registered dentist.

Sec. 9. That to provide a fund to carry out and enforce the provisions of this act the board of dental examiners may charge such fees, not exceeding one dollar for each certificate and ten dollars for each examination, as will from time to time, in the opinion of said board, approved by said Commissioners, be necessary. From such fund all expenses shall be paid by the board: *Provided*, That such expense shall in no case exceed the balance of receipts.

Approved, June 6, 1892.

